# Refining patient education on autologous chondrocyte implantation for chondral lesions of the knee: A fine‐tuned ChatGPT‐4o model improves readability and quality

**DOI:** 10.1002/jeo2.70445

**Published:** 2025-09-27

**Authors:** Maha Alsadaan, Stephen Fahy, Danko Dan Milinkovic, Benjamin Bartek, Tobias Winkler, Tobias Jung, Stephan Oehme

**Affiliations:** ^1^ Charité Universitätsmedizin Berlin, Centrum für Muskuloskeletale Chirurgie Berlin Germany; ^2^ Berlin Institute of Health at Charité Universitätsmedizin Berlin, Center for Regenerative Therapies Berlin Germany; ^3^ Berlin Institute of Health at Charité Universitätsmedizin Berlin, Julius‐Wolff‐Institute Berlin Germany

**Keywords:** autologous chondrocyte implantation, ChatGPT, DISCERN criteria, LLM, readability

## Abstract

**Purpose:**

Autologous chondrocyte implantation (ACI) is a complex procedure for cartilage defects, requiring patient understanding of treatment and recovery, as health literacy impacts outcomes. This study evaluated the quality and readability of AI‐generated ACI materials using ChatGPT‐4o as adjuncts to physician‐led education. We compared responses from the native model and a fine‐tuned version and hypothesised that the fine‐tuned model would provide improved quality and readability.

**Methods:**

Twenty‐two frequently asked questions were identified using Google's ‘People Also Asked’ feature. Two ChatGPT‐4o configurations were evaluated: the native model and a fine‐tuned version (ACI guide) optimised by instruction‐based fine‐tuning and reinforcement learning from human feedback. Two orthopaedic surgeons independently scored the responses. Quality was assessed using the DISCERN criteria and readability by the Flesch Reading Ease Score (FRES) and Flesch‐Kincaid Grade Level (FKGL). Interrater reliability was determined using intraclass correlation coefficient (ICC) in a two‐way mixed‐effects model.

**Results:**

The fine‐tuned ACI Guide outperformed the native ChatGPT‐4o on all parameters. The native model produced poor‐quality responses with a mean DISCERN score of 35.29 ± 5.0 (range: 23–45), while the ACI Guide achieved a significantly higher score of 43.18  ± 3.92 (range: 34–53; *p* < 0.001), reflecting moderate quality. Regarding readability, the native model reached FKGL of 13.45 ± 1.30 (university sophomore level)**.** In contrast, the ACI Guide achieved FKGL of 9.25 ± 1.64 (9th‐grade level). The FRES was also significantly higher for the ACI Guide (49.59 ± 10.44) than the native model (35.68 ± 5.08; *p* < 0.001). Interrater reliability was strong (ICC = 0.767), indicating good agreement.

**Conclusions:**

ChatGPT‐4o's responses were of poor quality and written at a readability level substantially exceeding recommended thresholds for patient education materials, limiting their applicability in clinical communication and patient education. Fine‐tuning ChatGPT‐4o improved the readability and quality of ACI patient education materials, generating content closer to the 8th–9th‐grade level. It may serve as a useful adjunct to physician‐led education in enhancing patient understanding of complex orthopaedic procedures.

**Level of Evidence:**

Level V.

**Abbreviations**
ACI: autologous chondrocyte implantation
ACL: anterior cruciate ligament
DISCERN: a standardised tool for assessing the quality of written health information
ESSKA: European Society for Sports Traumatology, Knee Surgery and Arthroscopy
FKGL: Flesch‐Kincaid Grade Level
FRES: Flesch Reading Ease Score
LLM: large language models
PAA: People Also Asked
PEMs: patient education materials
PRP: platelet‐rich plasma
RGL: reading grade level
RLHF: reinforcement learning from human feedback
SMOG: simple measure of gobbledygook
VPN: virtual private network

## INTRODUCTION

Cartilage lesions of the knee are a major concern in orthopaedics, often resulting in chronic pain, reduced mobility and progressive joint degeneration [15, 29]. In a retrospective study of 25,124 knee arthroscopies, chondral lesions were identified in 60% of the patients [28]. The regenerative capacity of articular cartilage is limited due to its low cell density and avascular, alymphatic nature. Consequently, autologous chondrocyte implantation (ACI) has emerged as a recognised therapy for symptomatic focal cartilage lesions [20, 21, 29]. ACI is a two‐stage procedure involving chondrocyte harvesting, in vitro expansion and re‐implantation after 5–6 weeks, followed by rigorous rehabilitation protocol [19, 29]. Due to the complexity of ACI, patients may struggle to understand the procedure and postoperative requirements, and limited health literacy has been shown to directly influence the patient's clinical outcomes, treatment adherence and overall satisfaction [4]. According to Rudd, traditional methods of patient education such as printed materials and in‐person consultations may either oversimplify the information or overwhelm patients with technical jargon, which ultimately hinders patients' ability to comprehend and engage with the material [23]. Consequently, some patients are turning to the internet as an additional source of information, spurred by the emergence of advanced AI tools such as ChatGPT. While these tools may enable access to information in a more accessible and personalised manner, they should be viewed as complementary to and not a substitute for professional medical advice and physician led education. However, this shift raises significant concerns about the readability and the quality of the information provided [7]. To achieve an average level of health literacy, patient education materials should be written at an American 8th to 9th‐grade reading level or lower [27]. However, this standard is frequently not met, particularly in digital health content, which often exceeds the recommended complexity for general audiences [9, 12]. This study assessed the potential of fine‐tuning ChatGPT‐4o to enhance patient health literacy in the context of ACI, by improving the quality and the readability of its responses compared to the native ChatGPT‐4o version. We hypothesise that the fine‐tuned version of ChatGPT‐4o will provide higher‐quality information with easier readability compared to the native ChatGPT‐4o, thereby making it a more effective tool for patient education.

## METHODS

### Question generation

The most commonly asked questions regarding ACI were generated using Google search engine (www.google.com) by entering the following keywords in the search tool: ‘Autologous chondrocyte implantation’, ‘ACI knee’ and ‘Autologous chondrocyte implantation (ACI) knee surgery’ on 01.02.2025. To minimise the impact of previous searched terms on our results, a new browser window and virtual private network (VPN) were used. After entering the keywords, Google generated a ‘People Also Asked’ (PAA) section for the searched topics, showing the most frequently asked questions by users related to each search term. This search yielded the top 27 user questions. After removing duplicates, a refined list of 22 commonly asked questions was finalised for analysis. The questions, provided by Google, were formulated to meet an 8th‐grade reading level. The reason for using the PAA feature is that it reflects real‐world patient search behaviour, as patients often turn to the internet to seek medical information [7]. Similar methods have been used in recent studies in orthopaedics [14, 17, 18, 25].

### ChatGPT‐4 configuration

In this study, we used two configurations of ChatGPT‐4o. The first configuration is the native ChatGPT‐4o version provided by OpenAI, without any additional fine‐tuning. The second configuration, ChatGPT‐4o, was fine‐tuned and named ‘ACI Guide’ to improve the information quality and to enhance the readability. The two configurations are described as follows:

### Native ChatGPT‐4o

This model is a general‐purpose configuration built to generate responses on a wide range of topics, utilising the ChatGPT‐4o architecture.

### Fine‐tuned ChatGPT‐4o (ACI guide)

To create this model, we used the following two methods: Instruction‐based fine‐tuning and reinforcement learning from human feedback (RLHF).

*Instruction‐based fine‐tuning*: The model was fine‐tuned using customised guidelines. This process involved directing the model to employ simple and direct language while steering clear of words containing two or more syllables. Additionally, it was instructed to avoid medical jargon and instead utilise terminology that is more widely recognised. These guidelines aimed at generating responses suitable for an 8th‐ and 9th‐grade reading level. To implement this, a dataset was created consisting of question–answer pairs written in accordance with these language rules.

For example, a training pair might include the question ‘What happens during ACI surgery?’ with the answer: ‘The doctor takes healthy cartilage cells from your knee, grows them in a lab, and puts them back into the damaged area to help it heal’.

*Reinforcement learning from human feedback (RLHF)*: This method enhanced the model by offering input on its outputs. The feedback cycle contributed to the model's clarity, readability and precision. Human evaluators played a crucial role in refining the model's responses for ACI, resulting in more accessible and more accurately cited outputs. Responses were generated and refined using the publicly accessible functionalities of ChatGPT‐4o Plus version. For example, when the model initially responded to the question ‘what are the risks of ACI?’ with ‘risks may include infection, graft failure, or persistent pain,’ reviewers noted the tone was too clinical and lacked explanation. Based on this feedback, the response was revised to be more patient‐friendly, including more available information and and reference to supporting information. This refinement process was repeated in several iterative cycles, with each round of feedback further enhancing clarity, accessibility and patient relevance.

### Data collection and analysis

The 22 questions were searched on 2 February 2025, using both the native ChatGPT‐4o and the fine‐tuned ‘ACI guide’. The resulting answers were then saved in a separate Microsoft Word document. This study did not involve human participants, patient data, or clinical interventions. Therefore, institutional review board (IRB) approval was not required.

### Quality assessment

The quality of the responses was assessed using the DISCERN criteria, a validated tool for assessing the quality of written consumer health information [6]. It consists of 16 questions rated on a scale from one to five. Questions 1–8 assess reliability, while questions from 9 to 15 focus on assessing the information provided about treatment options, including potential risks and benefits. Question 16 evaluates overall quality (see Table 1). The maximum score is 80. Scores ≥63 are considered ‘excellent,’ 51–62 ‘good,’ 39–50 ‘fair or moderate,’ and 27–38 ‘poor.’ Two orthopaedic surgeons with expertise in knee surgery (S.O. and S.F.) independently and blinded to the model version evaluated and scored the responses.

**Table 1:** The 16 DISCERN criteria used for quality assessment.

Section	Item	Criterion
Reliability	1	Are the aims clear?
	2	Does it achieve its aims?
	3	Is it relevant?
	4	Are sources of information clearly stated?
	5	Is it clear when the information was produced?
	6	Is it balanced and unbiased?
	7	Are additional sources of support/information provided?
	8	Does it refer to areas of uncertainty?
Treatment information	9	Does it describe how each treatment works?
	10	Does it describe the benefits of each treatment?
	11	Does it describe the risks of each treatment?
	12	Does it describe what would happen if no treatment is used?
	13	Does it discuss how treatment affects quality of life?
	14	Is it clear that multiple treatment choices may exist?
	15	Does it support shared decision‐making?
Overall assessment	16	Overall rating of the publication as a source of information

### Assessment of readability

To evaluate the text readability, the Readability Studio Professional Edition Program, Oleander Software Ltd., 2024, was utilised [22]. It provides a range of readability metrics and helps evaluate how easy or difficult a piece of writing is to comprehend. It incorporates several tests designed to analyse written material and recommend enhancements. In our study, we used the following two metrics: the Flesch–Kincaid Reading Grade Level (FKGL) and the Flesch Reading Ease Score (FRES) (see Table 2). The reported reading grade levels (RGLs) correspond to the grade level in the United States needed to understand the text. A text's difficulty is rated by FRES using a scale of 0–100. A score of 60 or higher is considered ‘plain English’, meaning that students between the ages of 13 and 15 could understand it with ease [11]. These readability scores have been validated and widely applied in orthopaedic literature to assess the complexity of both traditional and AI‐generated patient education materials [3, 8, 9, 10, 26].

**Table 2:** Summary of readability formulae.

Readability test	Score type	Formula	Description
Flesch–Kincaid reading grade level	Grade level	G = (11.8 × (B/W)) + (0.39 × (W/S)) ‐15.59	Developed for technical documents within the Kincaid Navy Personnel test series.
Flesch reading ease score	Index score (0–100)	I = (206.835 − (84.6 × (B/W)) − (1.015 × (W/S)))	A widely used test by U.S. government agencies for readability. Ideal for school textbooks and technical documents, it scores from 0 to 100, with higher values indicating easier readability.

*Note*: G = grade level; B = number of syllables; W = number of words; S = number of sentences; RGL = reading grade level; I = Flesch Index score.

### Statistical analysis

The Wilcoxon matched‐pairs signed‐rank test was used to assess statistically significant differences between the fine‐tuned and native ChatGPT‐4o outputs based on the mean total DISCERN score, the mean score per DISCERN category and readability metrics. To assess interrater reliability, the total DISCERN scores assigned by each evaluator across all 22 responses were analysed using a two‐way mixed effects intraclass correlation coefficient (ICC). IBM SPSS Statistics (version 30.0.0.0) was used for statistical analysis [16].

## RESULTS

### DISCERN score

**Figure 1:**
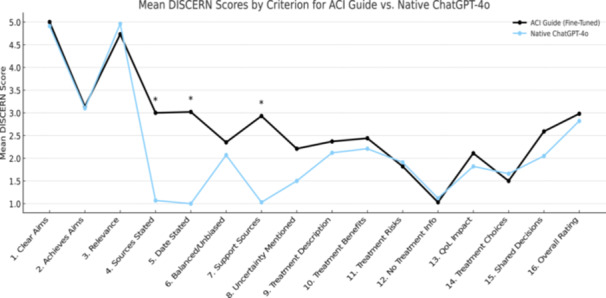
Mean DISCERN scores by criterion for the fine‐tuned ACI guide and native ChatGPT‐4o. ACI, autologous chondrocyte implantation.

The ACI Guide achieved a mean DISCERN score of 43.18 ± 3.92 (range: 34–53), reflecting moderate‐quality responses, while the native ChatGPT‐4o scored 35.29 ± 5.0 (range: 23–45; *p* < 0.001), indicating poor‐quality responses. Further analysis of the mean score of the responses of 22 questions in both versions for every individual DISCERN criteria confirmed that the fine‐tuned model achieved higher scores across nearly all 16 categories (Figure 1). The difference between the two versions was statistically significant. Additionally, the interrater reliability was assessed using a two‐way mixed effects ICC, yielding a value of 0.767, indicating good agreement between the evaluators.

### Readability

**Figure 2:**
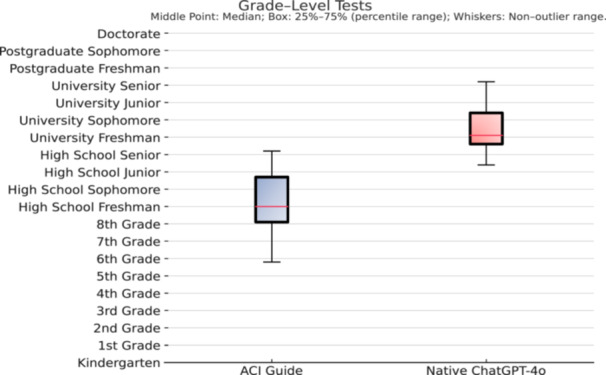
Box plot comparing the Flesch‐Kincaid readability levels of responses generated by native ChatGPT‐4o and the fine‐tuned ACI Guide. Boxes indicate the interquartile range (25th to 75th percentiles), with horizontal lines representing medians. Whiskers show the nonoutlier range. *Y*‐axis values correspond to U.S. educational grade levels. ACI, autologous chondrocyte implantation.

The ACI guide had a mean FKGL of 9.25 (range: 5.80–12.20, ±1.64), while the native ChatGPT‐4o had a significantly higher mean FKGL of 13.45 (range: 11.40–16.20, ±1.30) (*p* < 0.001) (see Figure 2). A lower FKGL score indicates greater readability, suggesting that the ACI Guide produced content requiring a 9th‐grade reading level, whereas the native model's content corresponded to a university sophomore reading ability. Similarly, the mean FRES score was significantly higher for the ACI Guide (49.59, range: 31–71, ±10.44) compared to the native ChatGPT‐4o (35.68, range: 27–48, ±5.08) (*p* < 0.001), reflecting greater ease of comprehension.

## DISCUSSION

The most important finding of this study is that fine‐tuning ChatGPT‐4o through instruction‐based methods and reinforcement learning from human feedback significantly enhanced the readability and quality of patient education materials on ACI. The fined‐tuned model referred to as the ‘ACI guide’ produced responses with higher quality and improved readability compared to the native ChatGPT‐4o. Instruction‐based fine‐tuning directed the model to respond more accurately to specific prompts, while reinforcement learning from human feedback ensures that the outputs are aligned with the expected standards [8].

The fine‐tuned ‘ACI guide’ generated responses of moderate quality, outperforming the native ChatGPT‐4o, which produced poor‐quality responses. The fine‐tuned model achieved higher scores across nearly all 16 DISCERN categories, with the most substantial improvement observed in source citation transparency, response's structural organisation, explanation of treatment benefits, and support for shared decision‐making. These specific categories are essential for ensuring that patients receive medical information that supports their autonomy and trust in the clinical decision‐making. However, it is important to note that AI‐generated responses should serve as an adjunct to and not a replacement for direct physician guidance. The surgeon remains central in personalising treatment options and counselling the patient. Our findings are consistent with prior studies conducted on ChatGPT‐4 as a patient potential educational tool for procedures such as high tibial osteotomy, where the fine‐tuned ChatGPT‐4 similarly demonstrated superior response quality compared to the poor‐quality responses of the native model [8]. A similar concept was applied in a customised model trained solely on the ESSKA osteotomy consensus, allowing the LLM to deliver responses that were better aligned with expert‐derived guidelines [24]. This use of domain‐specific training enabled the LLM to generate responses that were both more accurate and directly aligned with evidence‐based recommendations than those from the generic ChatGPT.

The readability was also substantially improved through ChatGPT‐4o fine‐tuning. Our ACI Guide achieved a FKGL of 9.25 and a FRES of 49.59, indicating greater ease of comprehension. This also aligns with the widely recommended standards that patient education materials (PEMS) should be written at an 8th to 9th‐grade level or lower to support adequate health literacy [27]. In contrast, the native ChatGPT‐4o generated content with a mean FKGL of 13.45 and a FRES of 35.68, indicating a reading difficulty equivalent to the university sophomore level, which substantially exceeds the accepted readability thresholds for patient educational materials. A high reading level typically involves longer sentences, advanced vocabulary and more complex sentence structures. For patients with limited literacy skills, this complexity can make it difficult to interpret and understand the health‐related information. Health literacy has been consistently identified as a key determinant of health outcomes and is regarded as one of the most robust predictors of an individual's health status, therefore it is essential to generate appropriate level of patient educational material [3].

Wang et al. demonstrated that existing online materials on the treatment of articular cartilage defects are written at an average FKGL of 13.4, a level that far exceeds the standard health literacy recommendation of an American 8th to 9th‐grade reading level or lower, which is consistent with our findings [26, 27]. This pattern is aligned with findings from studies evaluating native ChatGPT‐3.5 and ChatGPT‐4 across a range of topics including anterior cruciate ligament (ACL) injuries and platelet‐rich plasma (PRP) therapy, where outputs were classified as ‘difficult’ or ‘very difficult’ to read [9, 10]. Further supporting this pattern, a recent study by Chandra et al. found that the average readability of existing online materials for shoulder and elbow surgeries were written at a 10th‐grade reading level [5]. To address this, the researchers prompted ChatGPT‐4 to analysis the readability and to rewrite the content at a 6th to 8th‐grade level [18]. They assessed the generated materials further using the SMOG (Simple Measure of Gobbledygook) Index, a tool that estimates grade level based on the number of polysyllabic words in at least 30 sentences in total [5, 13]. As a result, their prompted ChatGPT‐4 has lowered the average SMOG Index from 10.1 to 8.3 and its own internal readability score from 10.3 to 7.7, bringing the content to the recommended 8th–9th‐grade reading level. This reduction of approximately two grade levels highlights the capability of LLMs to enhance the readability of complex medical information when properly guided. This finding is further supported by a study in the field of hand surgery, in which researchers prompted both ChatGPT‐4 and ChatGPT‐3.5 to generate simplified patient education materials at a 6th‐grade level, where ChatGPT‐4 outperformed ChatGPT‐3.5 in produced content at or below the 6th‐grade level [1]. In line with these observations, a study prompted four LLM (ChatGPT‐4, ChatGPT‐3.5, Claude 2 and Llama 2) to simplify orthopaedic patient education materials, while all models improved readability, only ChatGPT‐4 consistently reduced text to FKGL 6.72 while preserving accuracy and structure [2]. These findings collectively highlight the potential of LLMs to generate patient education materials that establishes the readability standards, particularly when guided through prompting or optimised through fine‐tuning. Fine‐tuned LLMs like the ACI Guide are designed to function as a supportive tool that complements physician‐based education. In real‐world settings, it could be used to provide patients with easy‐to‐understand information before or after consultations, clarify postoperative instructions, or reinforce key points discussed during informed consent. By enhancing the readability and quality of educational content, fine tuned LLMs like the ACI Guide could help patients better comprehend their condition and treatment options, ultimately supporting shared decision‐making and improving communication between patients and healthcare providers.

Our study faced several limitations that must be acknowledged. Starting with the evolving nature of ChatGPT, prior research has demonstrated performance differences between several versions, with ChatGPT‐4 generally outperforming ChatGPT‐3.5 [9, 10]. Second, we used the DISCERN criteria to evaluate AI‐generated responses, while DISCERN is a validated and widely accepted instrument for assessing the quality of traditional patient education materials, its applicability to AI‐generated content has not yet been formally validated [6]. Nevertheless, it remains a useful and structured framework for assessing key aspects of information quality. Future studies may benefit from adapted or AI‐specific evaluation tools as the field evolves. Additionally, this study focused on a single clinical topic ‘ACI’, which may limit the generalisability of our findings. Despite these limitations, our findings provide timely and relevant evidence that targeted fine‐tuning can substantially improve the quality and readability of ChatGPT‐4o generated patient education materials in the context of ACI.

## CONCLUSION

Fine‐tuning ChatGPT‐4o significantly improved the quality and readability of patient education materials related to ACI. This represents a clear improvement over the native model, whose responses were often sparsely referenced and exceeded readability recommendations. These gains required minimal fine‐tuning, achieved through instruction‐based modifications and human feedback. Given ACI's complexity and the role of health literacy in shared decision‐making, this approach shows promise as an adjunct to physician‐led education. Future studies should explore its integration into ACI education and broader orthopaedic use.

## AUTHOR CONTRIBUTIONS

**Maha Alsadaan**: Data curation; statistical analysis; visualisation; software; writing (original draft, review and editing). **Stephan Oehme**: Writing (original draft, review and editing). **Stephan Oehme** and **Stephen Fahy**: Conceptualisation and DISCERN scoring. **Danko Dan Milinkovic** and **Stephen Fahy**: Writing (review and editing). **Tobias Jung, Tobias Winkler** and **Benjamin Bartek**: Supervision of the study and revision of the manuscript. All authors confirm their approval of the final version of the manuscript.

## CONFLICT OF INTEREST STATEMENT

The authors declare no conflict of interest.

## ETHICS STATEMENT

This study did not involve human participants, animals, or patient data. Therefore, ethical approval was not required.

## Supporting information

Supplementary. Most frequently asked ACI‐related questions generated through Google's *“People Also Asked”* (PAA) feature.

## Data Availability

The datasets used and analysed during the current study are available from the corresponding author upon request.
