# Supplementary material for: Refining patient education on autologous chondrocyte implantation for chondral lesions of the knee: A fine‐tuned ChatGPT‐4o model improves readability and quality
Source: J Exp Orthop. 2025 Sep 27;12(4):e70445. doi: 10.1002/jeo2.70445 (PMC12475932; doi:10.1002/jeo2.70445)
Supplement: Supplementary file 1 — Supplementary. Most frequently asked ACI‐related questions generated through Google's “People Also Asked” (PAA) feature. [file JEO2-12-e70445-s001.docx]

ACI Question’s List

| **1** | What is ACI therapy for knees? |
| --- | --- |
| **2** | Can chondrocytes regenerate cartilage? |
| **3** | Can you fix cartilage without surgery? |
| **4** | What are the indications for ACI? |
| **5** | Who is the ideal candidate for autologous chondrocyte implantation? |
| **6** | What is ACI for knee arthritis? |
| **7** | What is the first stage of ACI? |
| **8** | How long does ACI surgery take? |
| **9** | How long does it take to grow knee cartilage in a lab? |
| **10** | Does cartilage grow back after surgery? |
| **11** | What is the recovery time for ACI knee surgery? |
| **12** | What is rehabilitation after autologous chondrocyte implantation? |
| **13** | What is the success rate of autologous chondrocyte implantation? |
| **14** | Is ACI therapy effective? |
| **15** | What is the failure rate of ACI? |
| **16** | Can your body reject a cartilage transplant? |
| **17** | How long do cartilage transplants last? |
| **18** | What are the complications of autologous chondrocyte implantation? |
| **19** | How much does ACI knee surgery cost? |
| **20** | What is the difference between ACI and OATS knee? |
| **21** | Is ACI better than Mosaicplasty? |
| **22** | Is a cartilage transplant better than a knee replacement? |
|  | Flesch-Kincaid Reading Grade Level: 8.1 |
|  | Gunning Fox Index: 11.4 |
|  | Flesch Reading Ease: 58 |
